# Virtual conferences raise standards for accessibility and interactions

**DOI:** 10.7554/eLife.62668

**Published:** 2020-11-04

**Authors:** Sarvenaz Sarabipour

**Affiliations:** Institute for Computational Medicine, Department of Biomedical Engineering, Johns Hopkins UniversityBaltimoreUnited States

**Keywords:** virtual conferences, science communication, education, research culture, diversity, early-career researchers, None

## Abstract

Scientific conferences have an important role in the exchange of ideas and knowledge within the scientific community. Conferences also provide early-career researchers with opportunities to make themselves known within their field of research. Although the COVID-19 pandemic has brought traditional in-person conferences to a halt for the foreseeable future, the growth of virtual conferences has highlighted many of the disadvantages associated with the in-person format and demonstrated the advantages of moving these events online. Here, based on data from in-person and virtual conferences in a range of subjects, we describe how virtual conferences are more inclusive, more affordable, less time-consuming and more accessible worldwide, especially for early-career researchers. Making conferences more open and inclusive will provide both immediate and long-term benefits to the scientific community.

Many researchers are unable to attend in-person conferences due to financial and logistic barriers (Sarabipour et al., 2020; Figure 1). Virtual conferences lower or remove these barriers by reducing both costs and travel times: they also reduce the 'red tape' (e.g. the need for visas) experienced by some researchers, and make it easier for those with disabilities or vulnerabilities and those with caring responsibilities to take part. Another advantage is that they have a much reduced carbon footprint (Sarabipour et al., 2020). Virtual conferences are also significantly cheaper for scientific societies to organize (Castelvecchi, 2020), allowing lower registration costs than in-person meetings. This enables a more efficient use of funding, since attending in-person conferences costs researchers an aggregate of tens of billions of dollars annually (Sarabipour et al., 2020; Row, 2019). Largely due to the COVID-19 pandemic, the number of online conferences has increased in 2020, attracting large numbers of participants worldwide (Figure 1, Figure 1—figure supplements 1 and 2).

**Figure 1.**
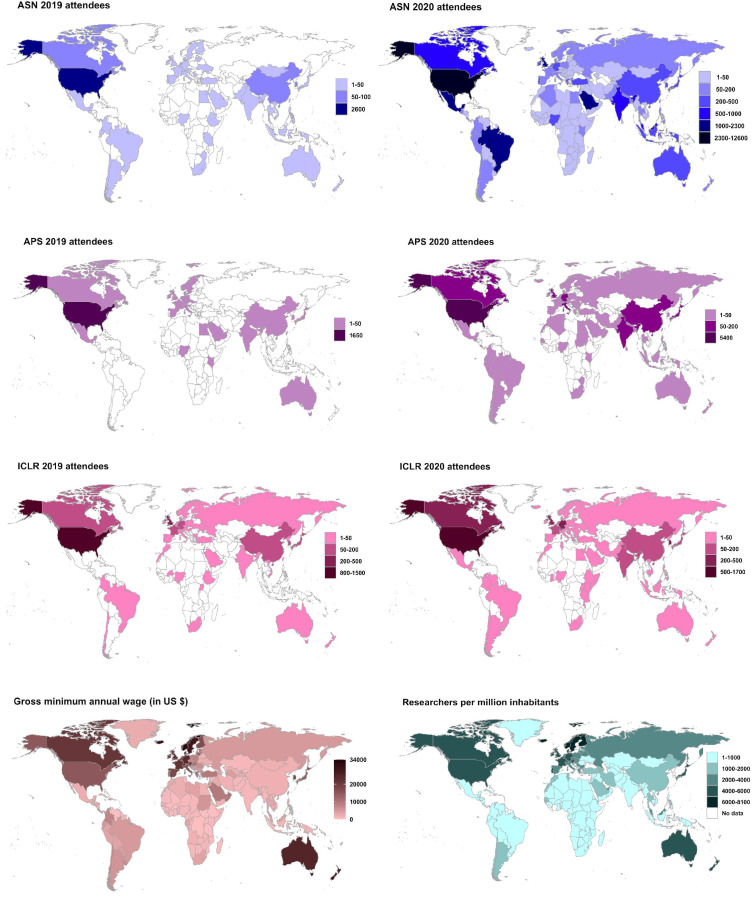
The fully virtual conferencing mode has been successfully adopted by scientific societies and researchers across the globe. The fully virtual meeting structure has enabled record attendance and geographic, socioeconomic and career stage diversity of attendees, an unprecedented level of human interactions, and exchange of scientific ideas and discussions online. From top left to right: The Nutrition Science meeting (Nutrition 2019), the annual meeting American Society for Nutrition (ASN), was held in-person with 3,157 attendees from 59 countries. Nutrition 2020 was hosted online with free registration, financially supported by the ASN foundation and had over 30,000 participants from 167 countries. The 2019 American Physical Society April meeting (APS 2019) was held in person with 1,758 attendees, while APS 2020 was held virtually and had 7,267 participants worldwide. The 7th international conference on learning representations (ICLR 2019), one of the world’s major machine learning conferences, welcomed a total of 2,528 attendees from 50 countries in person in New Orleans, Louisiana. The 8th international conference on learning representations (ICLR 2020), was held virtually with a total of 4,583 attendees, and 1,336 speakers participated virtually from 89 countries. This virtual meeting also boasted over 100,000 chat messages, 100,000 video watches, 200 unique average views per page and 1 million total page views and time spent at sponsor booths (ICLR Organizing Committees, 2020). ICLR 2020 had no registration fees. Aggregate anonymous attendee counts and represented countries were courtesy of conference organizers and scientific societies (http://doi.org/10.5281/zenodo.4044404). Last two panels: the gross (full amount an employer pays before taxes and other deductions are withheld) minimum annual (12 months) wages worldwide (in US dollars). The salary data shown are for the year 2019 (data source https://data.worldbank.org/indicator/PA.NUS.PRVT.PP). Early-career researcher salaries are close to the minimum annual wage worldwide (data source: https://www.payscale.com/research/UK/Job=Postdoctoral_Research_Associate/Salary). Attending a single national or international conference typically costs USD $1,000–4,000 (data source: https://elifeambassadors.github.io/improving-conferences/) thus attending in-person conferences is not feasible for many researchers, especially early-career researchers worldwide. The last panel shows the latest (2018) number of researchers per million inhabitants worldwide. Researchers (in full-time equivalent) per million inhabitants is a direct measure of the number of research and development workers per million people. (data source: https://en.unesco.org/node/252277). The world average number of researchers per million inhabitants is ~1,345. The educational, career development and networking opportunities offered by virtual conferences to a larger population of researchers can in the short-term enhance the visibility of under-privileged researchers, and in the long-term help improve researcher density for hundreds of nations with low numbers of researchers.

**Figure 1—figure supplement 1.**
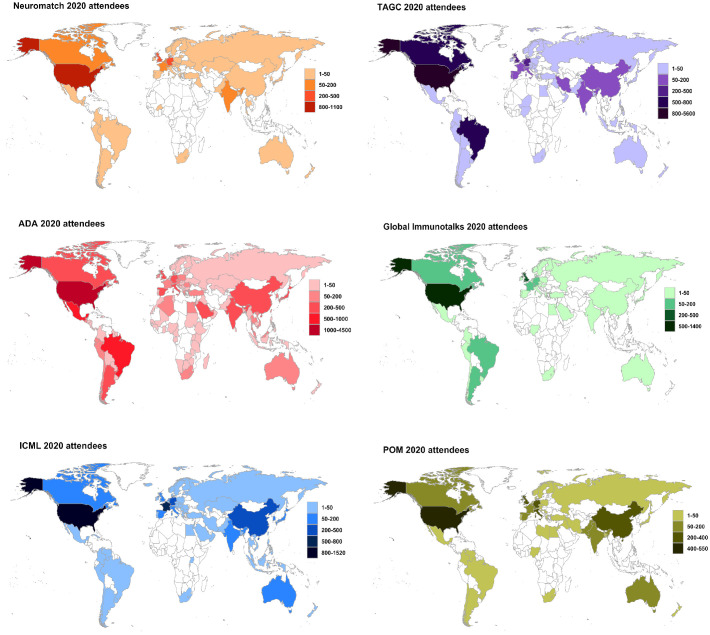
Attendees to six virtual conferences in 2020 by country. From left to right and top to bottom: number of attendees coming from each country to Neuromatch 2020, TAGC 2020, ADA 2020, Global Immunotalks 2020, ICML 2020 and POM 2020. The intensity of the colors represents the number of attendees as indicated by each colorbar.

**Figure 1—figure supplement 2.**
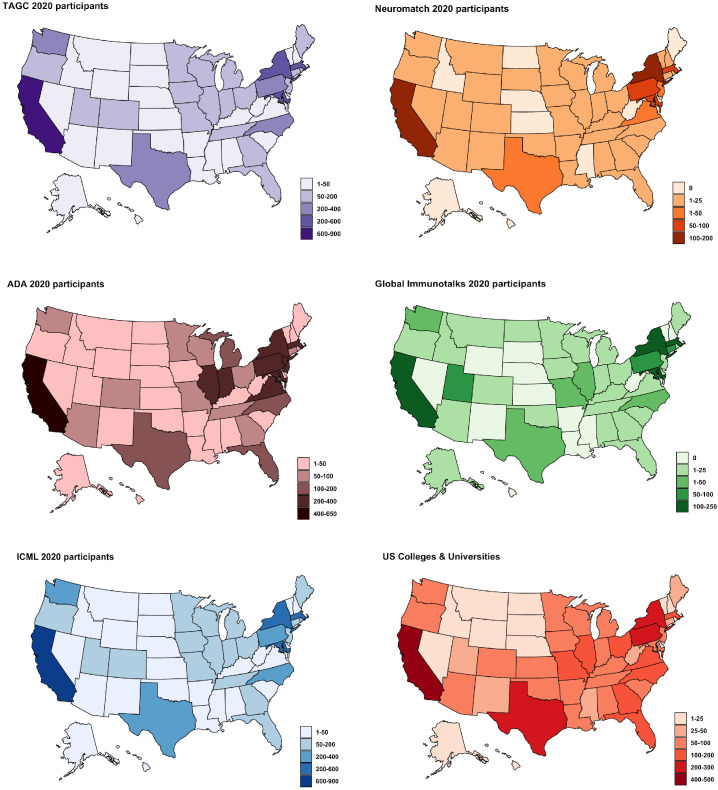
Attendees to five virtual conferences in 2020 by state (in the United States) and number of colleges and universities by state. From left to right and top to bottom, maps of the USA showing the number of attendees from each state in the United States to TAGC 2020, Neuromatch 2020, ADA 2020, Global Immunotalks 2020 and ICML 2020; the number of accredited academic institutions per state is shown in the bottom right panel.

Virtual formats have improved conferencing using a host of audio-visual technologies to facilitate real-time talks, extended Q and A sessions, electronic posters and follow up discussions, training workshops, informal networking, brainstorming events and virtual industry exhibits. A number of conferences have incorporated virtual reality tools to create new conference environments, and apps and machine learning algorithms to match attendees of similar research interests into virtual discussion rooms, enabling networking and collaboration (Achakulvisut et al., 2020; ICLR Organizing Committees, 2020). Some virtual conferences were held for the extended timeframes (weeks instead of days) enabling participation of researchers from multiple time zones via live and recorded talks, asynchronous discussions and social meetups. Recordings allowed talks to be paused or rewound, a useful feature for those who missed details or planned to spend more time pondering a crucial slide. Virtual conferences have further increased structured archiving of and open access to abstracts, posters and other research materials. Live review and analysis of research presentations and other scientific outputs enabled wide ranging engagement between speakers and audiences globally (reaching 100,000 chat messages at a single conference).

Virtual conferences can help researchers and scientific societies to meet more frequently as well and build long-term, inclusive, economically sustainable, and easily accessible communities nationally and globally in specific disciplines and across disciplines.

## Fostering the participation of underrepresented researchers in conferences

In-person conferences exasperate inequalities in academic communities (Sarabipour et al., 2020). Increasing the diversity of research environments is vital for scientific innovation and will increasingly be the focus of research and funding institutions (ERC, 2020; Wellcome, 2020).

Undergraduate trainees, graduate students, postdoctoral researchers, and female academics have fewer economic means and travel less than tenured professors and male colleagues to attend in-person conferences (Wynes and Donner, 2018; Jöns, 2011). Since virtual meetings have lower or no registration fees, they are open to a much wider variety of researchers. Having researchers from diverse backgrounds and life experiences at scientific meetings brings more varied perspectives and creativity towards addressing complex scientific problems.

Enabling presentations in multiple languages with captioning and translation will further encourage scientific exchange across nations. Additionally, providing closed captions and transcripts of virtual talks will further assist attendees with hearing and visual disabilities.

**Figure 2.**
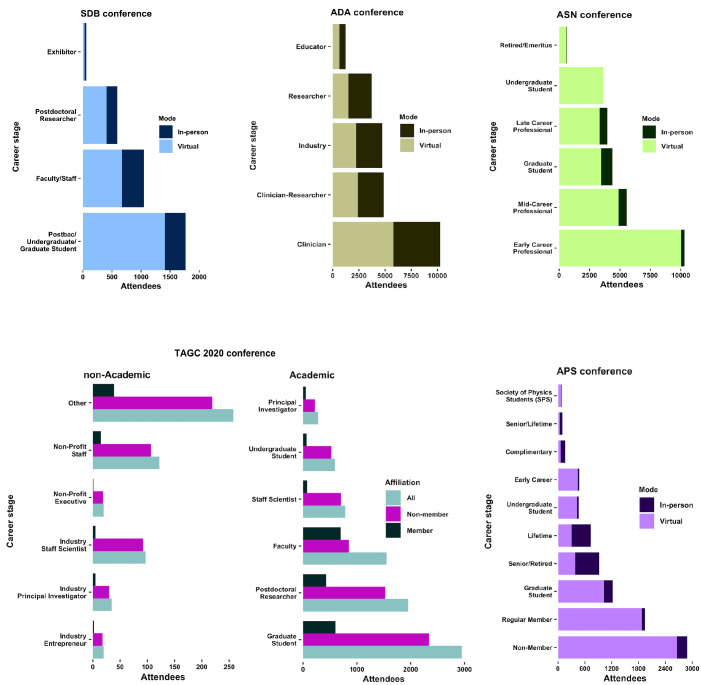
The virtual mode and lower attendance and registration costs enable a record high attendance of all career stages and research sectors. The fully virtual meeting structure has enabled much higher attendance of researchers from diverse career stages, notably early-career researchers (ECRs) (trainees and faculty). From top to bottom and left to right: The Society for Developmental Biology (SDB) annual meeting was attended by 949 researchers in-person in 2019 and 2480 researchers virtually in 2020. The virtual meeting was held at a reduced registration cost. The American Diabetes Association (ADA) annual meeting was held in-person in 2019 and virtually in 2020. Attendance at ADA 2020 required a registration fee but ~1,000 more clinicians attended the ADA 2020 virtual meeting compared to the in-person ADA 2019. The American Society for Nutrition (ASN) meeting was held virtually in 2020 free of registration costs. The American Physical Society (APS) 2019 meeting was held in-person and the 2020 meeting was held virtually. The Allied Genetics Conference (TAGC), a meeting of the Genetics Society of America (GSA), has only been held twice (in-person in 2016 and virtually in 2020). TAGC 2020 virtual conference attendance required no registration fee hence experienced a very high level of GSA member and non-member participation at all career stages and research sectors (academia, non-profit and industry). ‘Other’ in all panels may include private citizens.

**Figure 3.**
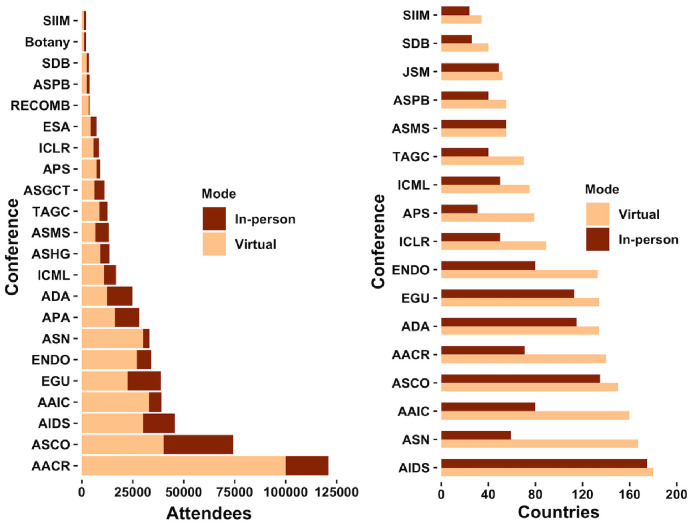
Virtual conferences enable a multiple-fold increase in attendance from more countries. (Left) Total number of attendees, and (right) total number of countries the attendees participated from. Shown are the typical in-person (year 2019) and 2020 virtual attendee counts for annual meetings of American Association for Cancer Research (AACR), American Society of Clinical Oncology (ASCO), European Geosciences Union (EGU), AIDS biennial conference, The Endocrine Society (ENDO), American Society for Nutrition (ASN), American Diabetes Association (ADA), Alzheimer's Association International Conference (AAIC), American Society for Human Genetics (ASHG), American Society for Mass Spectrometry (ASMS), American Society for Plant Biology (ASPB), International Conference for Learning Representation (ICLR), International Conference for Machine Learning (ICML), Research in Computational Molecular Biology (RECOMB), Society for Developmental Biology (SDB), the quadrennial meeting of the Genetics Society of America (TAGC), Joint Statistical Meetings (JSM) of the American Statistical Association, Society for Imaging Informatics in Medicine (SIIM), American Physical Society (APS) April meeting, the Ecological Society of America (ESA), the American Botanical Society meeting (Botany), American Society for Gene and Cell Therapy (ASGCT), American Psychological Association (APA). The American Association for Cancer Research (AACR) annual conference is attended by less than 24,000 scientists and clinicians in all subdisciplines of cancer research in-person every year. The AACR 2020 meeting took place in two parts virtually with over 100,000 attendees (Virtual Meeting I (April 27–28)) and (Virtual Annual Meeting II (June 22–24)) combined from 140 countries. At peak sessions, 24,000 attendees viewed the talks at the same time. Attendance at AACR 2020 was free for all (no registration fee for researchers or non-researchers). Available data on attendees and representing countries for in-person conferences held in years prior to 2019 are shown in Figure 3—figure supplement 2.

**Figure 4.**
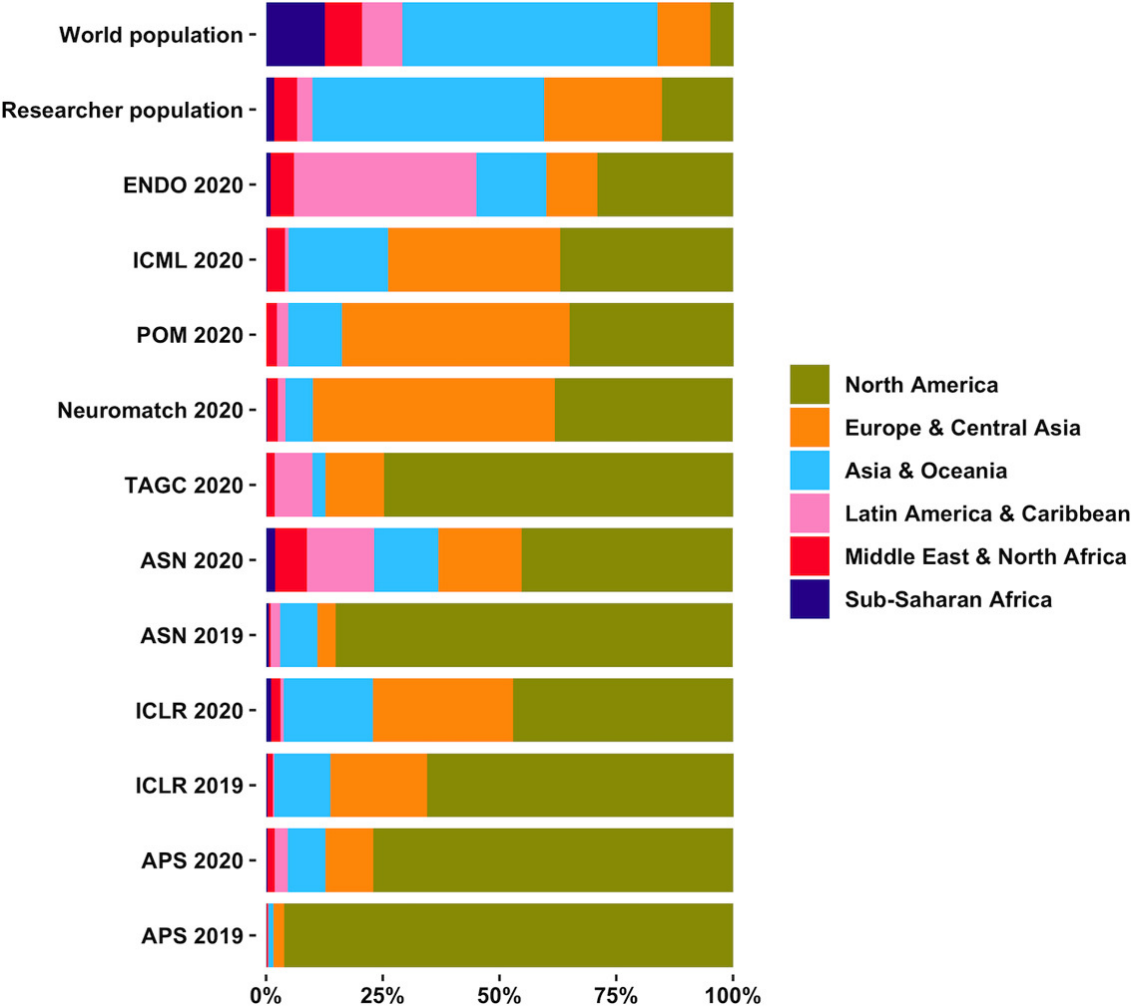
Virtual conferences are more inclusive and help improve the geographic diversity of attendees. Total continent population as a percentage of the world population, total continent full time or equivalent researcher population as a percentage of world full time or equivalent researcher population (~9.94 million) as of 2018 (data source: https://en.unesco.org/node/252277), and in-person (2019) and virtual (2020) meeting attendee continental distributions are shown for the annual meetings of the International Conference for Learning Representation (ICLR), the April meeting of American Physical Society (APS), the American Society for Nutrition (ASN), the Endocrine Society annual meeting (ENDO), the Photonics Online Meetup (POM), Neuromatch, the Allied Genetics Conference (TAGC), International Conference for Machine Learning (ICML). The APS 2019, ASN 2019, ICLR 2019 and ENDO 2020 meetings were held at full registeration cost of in-person meetings. The TAGC 2020, POM 2020, ASN 2020, ICLR 2020, APS 2020, ICML 2020 and Neuromatch 2020 meetings were held at low registeration cost or free of registration cost for all.

Online conferences have allowed much higher levels of participation of researchers from all scientific sectors internationally – and, in particular, researchers from underrepresented groups, researchers from countries with low-to-middle income economies, and early-career researchers (Figure 1, Figure 1—figure supplement 1; Figure 2; Figure 3; Figure 4). Well-planned virtual conferences can also significantly increase the diversity of speakers, increase representation of historically marginalized researchers, and help to achieve gender parity (De Saá‐Pérez et al., 2015). A higher number of attendees also means presentation of diverse research, viewpoints, discussions and ideas, which will benefit all researchers, since diversity brings excellence to scientific exchanges (De Saá‐Pérez et al., 2015; Nielsen et al., 2018).

**Figure 2—figure supplement 1.**
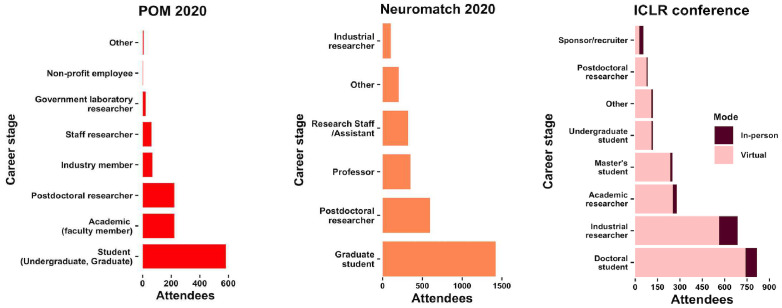
Attendees to three virtual conferences in 2020 by career stage. From left to right, attendees to POM 2020, Neuromatch 2020 and ICLR by career stage. For ICLR both in-person (2019) and virtual (2020) numbers are shown.

**Figure 3—figure supplement 1.**
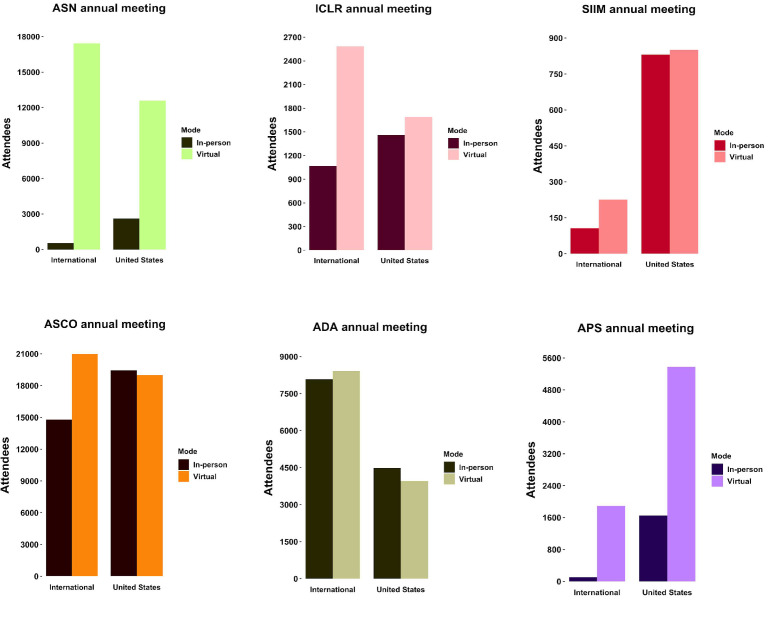
Attendance to six conferences in either their in-person or virtual formats, showing both international and US attendance. From left to right and top to bottom, attendance to the ASN annual meeting, the ICLR annual meeting, the SIIM annual meeting, the ASCO annual meeting, the ADA annual meeting and the APS annual meeting.

**Figure 3—figure supplement 2.**
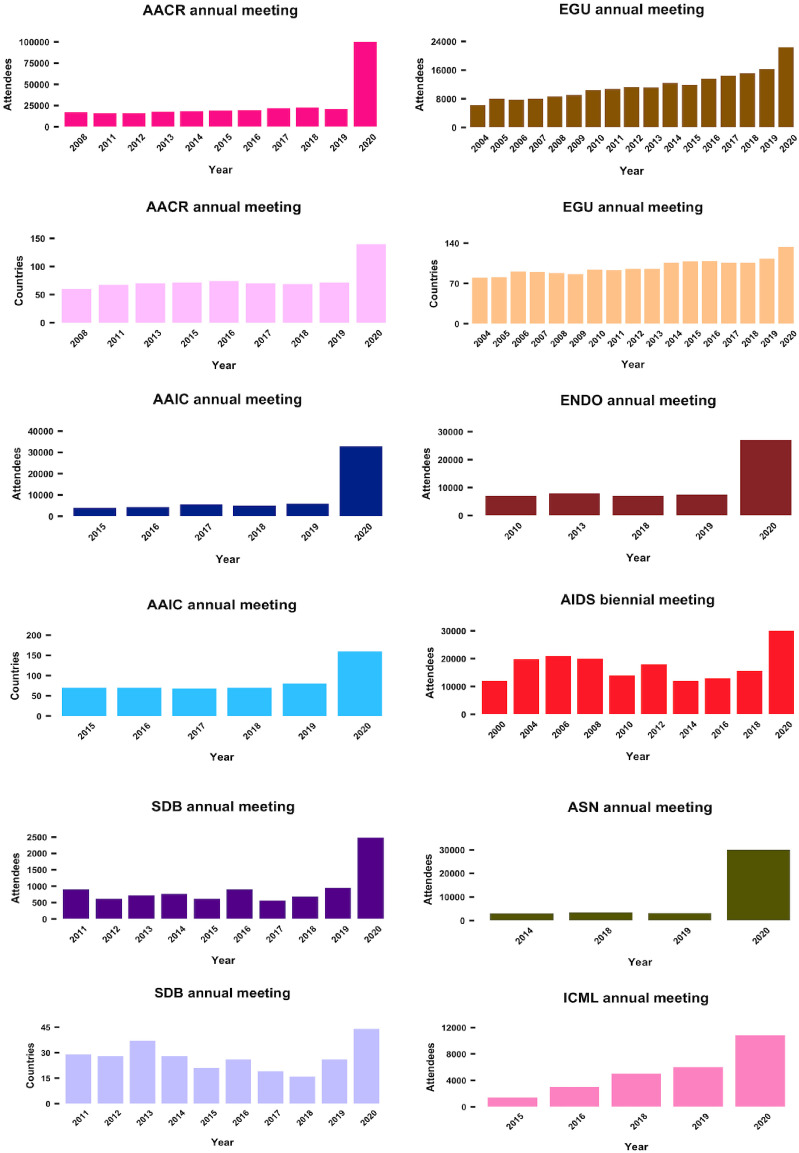
Change in attendance by number of participants and country of residence depending on the year for eight conferences that have switched to a virtual format in 2020. From left to right and top to bottom, participants in the AACR annual meeting, participants in the EGU annual meeting, countries of residence of participants in the AACR annual meeting, countries of residence of participants in the EGU annual meeting, participants in the AAIC annual meeting, participants in the ENDO annual meeting, countries of residence of participants in the AAIC annual meeting, participants in the AIDS biennial meeting, participants in the SDB annual meeting, participants in the ASN annual meeting, countries of residence of participants in the SDB annual meeting, and participants in the ICML annual meeting,.

Unfortunately, conferences in many disciplines are still heavily dominated by speakers who are male and/or from economically well-off nations (Sarabipour et al., 2020). Co-organizing conferences and delivering conference talks, particularly as an invited, featured, plenary or keynote speaker, are major career milestones that enhance the national and international scientific profile of any researcher. As such, passing over women and other underrepresented researchers for speaking and organizing engagements harms their careers. Concerted efforts are required to create equity for attendees in featured speakers, session chairs, and scientific and organizing committee member roles (Sarabipour et al., 2020). If representation is not increased at these positions and opportunities and the current culture persists, parity at funding rates, award nominations, early-career researcher (ECR) hiring patterns or promotion from junior to senior faculty levels and fair treatment of women will be less likely in the long term. Online conferences provide an unparalleled opportunity to increase representation.

## Acceleration of knowledge transfer, scientific innovation and progress

Virtual conference platforms can provide better file sharing, data presentation, and interactive visualization formats for the speakers and attendees. For instance, the 2020 International Conference on Learning Representations provided interactive figures and animations (ICLR Organizing Committees, 2020), which are often not supported by the platforms used to host conference abstracts or peer-reviewed articles. The virtual format also allows participants to comment on and exchange computer code, workflows, and other interim research products while the conference is ongoing. Digital libraries of the abstracts, talks, slides, and posters further provide the opportunities to attend and watch the entire conference including parallel oral and poster sessions. This ability to share and present data more efficiently will accelerate scientific progress as researchers can immediately access new information that they can use in their own research.

Further, virtual conferences attract record numbers of researchers from all continents and time zones (Figure 1, Figure 1—figure supplement 1, Figure 3, Figure 3—figure supplements 1 and 2). Researchers who have to cope with socioeconomic, geographic and/or physical limitations, and researchers with family commitments and responsibilities can all attend virtual events. Additionally, low or nil registration costs have meant that more researchers can attend multiple virtual conferences per year. This increases the number of people with access to new research and ideas, which will lead to more innovation in more countries compared to in-person conferences.

## Promotion, training and sponsorship opportunities for early-career researchers

The open and global setting of virtual conferences enables wide inclusion of demographics and productive discussions that can lead to long-term collaborations among researchers of different career stages. The virtual setting also allows for better training opportunities for ECRs, by increasing opportunities to present their research.

Low registrations costs enable a higher number of ECRs to attend virtual conferences (Figure 2, Figure 2—figure supplement 1), allowing them to take advantage of interactions with more senior researchers. These interactions can lead to collaborations and/or to learning about potential studentships, internships, postdoctoral opportunities or faculty positions.

Well-planned conferences need a good ratio of early-career researchers to senior scientists acting as speakers or session chairs. In in-person conferences, this often means that not everyone who submits a quality abstract can give a talk, and it is usually ECRs who miss out on valuable speaking time and exposure. Virtual conferences can solve this issue by allowing participants to watch talks asynchronously or by being organized over a period of time, which allows most submitted abstracts to be accepted for oral presentations.

Besides increasing scientists’ opportunities to participate in conferences, online discussion platforms during conferences can also help establish long-term collaborations and mentorship relationships between scientists in different career stages, including employment and sponsorship opportunities for ECRs.

Finally, peer-mentorship and collaborative peer-review of conference abstracts and other research products during virtual conferences will increase discussion and transparency on research, potentially improving the reproducibility of the research presented.

## Conferences organizers need to release meeting statistics

Virtual conferences allow organizers to gather participant feedback and data on demographics, audience engagement and levels of networking, which would be difficult or impossible to track during in-person meetings.

This information, along with data on attendee and speaker career stage and awardee and honoree status is highly valuable (Le et al., 2020) and can be used to improve conferences. A few steps to do this are: (i) conducting pre- and post-conference surveys to receive attendee feedback; (ii) reporting speaker line-ups; (iii) enabling diversity data to be self-reported by attendees; (iv) reporting statistics about the demographics of speakers to assess equity, inclusion and diversity (EDI); and (v) using anonymized data to uncover specific diversity baselines and act to increase EDI.

These steps will help conferences to: (a) better understand research communities in specific disciplines; (b) set and share policies to improve EDI practices across multiple continents; (c) address disparities in gender, country of affiliation, ethnicity and race, language and career stage; (d) assess connections, networking and collaborations; and (e) evaluate the impact of academic gatherings on research and research culture.

Improvements do not have to rely just on self-evaluation: conferences can also influence each other. Accessibility solutions, lessons learned, and improvements in policies at one conference and country can be shared with and adopted by other meetings, making it essential for conference organizers to transparently share policies, measurements, language, and standards.

## Conclusions

Science is a global endeavor. Academia must recognize the professional privilege required to attend in-person conferences, and reformulate policies, attitudes, and funds towards organizing virtual conferences with a view toward achieving an equitable system for women, minorities and all researchers worldwide.

The shift to virtual conferencing prior to, and increasingly during, the COVID-19 pandemic has provided an unprecedented opportunity to reform methods of organizing academic conferences in all disciplines. The potential benefits of scientific conferences have been underutilized in the in-person format, and can be further explored in virtual formats. This year, researchers from around the globe and across the full spectrum of research and career stages gained access to innovative research and opportunities to engage and connect. The fully virtual meeting structure enabled unprecedented levels of interactions and exchange of scientific ideas and discussions online.

It is crucial that scientific societies and conference organizers examine the data generated, the actions taken and the outcomes of conferences, and then pool resources from private and public funding organizations to further improve these gatherings and accelerate changes in research culture. To do this, it is particularly important to consider the needs and constraints of meeting participants, and ECRs in particular. These efforts will have both short- and long- term impact on research and research culture. Curating the outputs of conferences (such as code, protocols, workflows, abstracts and manuscripts) in open digital libraries will increase access to scientific research. A culture of open digital communication, group reviews and live national and international discussions will strengthen scientific bonds among researchers in all disciplines. It can also help to improve transparency and reproducibility of research findings to accelerate the rate of scientific and medical discoveries. Broad and speedy access to scientific knowledge worldwide will improve research, health, environmental and economic conditions in all countries for years to come.

## Methods

The data used for visualization was pulled from multiple sources. Existing data on in-person conferences were sourced from the internet via an in-person conference database curated by the author at https://elifeambassadors.github.io/improving-conferences/, the conference and respective scientific society websites, and Google search. Aggregate number of attendees and representing countries were collected for the years that data was available. Data on country specific and state specific attendance was requested for in-person conferences of 2019 and virtual conferences of 2020 from scientific societies and in a number of cases were provided to the author. Data on the 2019 gross minimum wage (full amount an employer pays before taxes and other deductions are withheld) minimum annual (earned for 12 months in US dollars) wages worldwide was sourced from the world bank database at https://data.worldbank.org/indicator/PA.NUS.PRVT.PP. The latest available data on the number of researchers per million inhabitants worldwide was sourced from the UNESCO statistics institute database (https://en.unesco.org/node/252277). The number of accredited academic institutions in the United States was sourced from (https://catalog.data.gov/dataset/colleges-and-universities-cb8a4). A table with the acronyms for the different conferences discussed in the article is available in Supplementary file 1.

## Visualization

All figures were made with R and the ggplot2 package (Wickham, 2016), with colors from the RcolorBrewer package (Neuwirth, 2014). Code and aggregate anonymous raw data on attendee counts and represented countries and career stage statistics underlying all figures is available on (http://doi.org/10.5281/zenodo.4044404).

## Data Availability

Code and aggregate anonymous raw data on attendee counts and represented countries and career stage statistics underlying all figures is available on (http://doi.org/10.5281/zenodo.4044404). The following dataset was generated: SarabipourS2020Analysis of Virtual ConferencesZenodo10.5281/zenodo.4044404

## Additional files

Supplementary file 1. Acronyms and abbreviations. Table including the acronyms and abbreviations for all scientific meetings used in the article and where each conference was held.
